# Assessing axitinib-induced differential responses in tumor vascularization and oxygenation with combined optoacoustic angiography and diffuse optical spectroscopy

**DOI:** 10.1016/j.neo.2026.101303

**Published:** 2026-04-10

**Authors:** Anna Orlova, Ksenia Akhmedzhanova, Anna Glyavina, Alexey Kurnikov, Dmitry Khochenkov, Yulia Khochenkova, Artemii Korobov, Artur Volovetskiy, Andrey Yudintsev, Anastasiya Nerush, Anna Maslennikova, Vladimir Vodeneev, Ilya Turchin, Wei Liu, Daniel Razansky, Pavel Subochev

**Affiliations:** a Department for Radiophysical Methods in Medicine, A.V. Gaponov-Grekhov Institute of Applied Physics RAS, Nizhny Novgorod, Russia; b Department of Biophysics, Institute of Biology and Biomedicine, Lobachevsky State University of Nizhny Novgorod, Nizhny Novgorod, Russia; c Laboratory of Biomarkers and Mechanisms of Tumor Angiogenesis, Research Institute of Experimental Diagnostics and Therapy of Tumors, N.N. Blokhin National Medical Research Center of Oncology, Moscow, Russia; d Center for Medical Chemistry, Institute of Chemistry and Energy, Togliatti State University, Togliatti, Russia; e Center for Photonic Science and Engineering, Skolkovo Institute of Science and Technology, Moscow, Russia; f Laboratory of Molecular Pharmacology, Institute of Molecular Theranostics, I.M. Sechenov First Moscow State Medical University of the Ministry of Health of the Russian Federation, Moscow, Russia; g Optical Imaging Laboratory, Harbin Institute of Technology, Shenzhen 518055, China; h Institute for Biomedical Engineering and Institute of Pharmacology and Toxicology, Faculty of Medicine, UZH Zurich, Zurich, Switzerland; i Institute for Biomedical Engineering, Department of Information Technology and Electrical Engineering, ETH Zurich, Zurich, Switzerland

**Keywords:** Tumor angiogenesis, Tumor oxygenation, Axitinib, Optoacoustic imaging, Diffuse optical spectroscopy, Experimental tumor

## Abstract

Efficacy of antiangiogenic treatments is often linked to the complex interplay between tumor vascularization and oxygenation. Yet their relationship remains difficult to assess *in vivo* due to limitations of conventional clinical imaging techniques. We used a combination of noninvasive optoacoustic (OA) angiography and diffuse optical spectroscopy (DOS) to investigate the effects of the antiangiogenic therapy on vascular structure and oxygenation in subcutaneous xenograft model of Colo320 colon adenocarcinoma. Axitinib, a tyrosine kinase inhibitor targeting VEGF receptors, was administered into animals at 50 mg/kg, five days per week for four weeks. Raster-scan OA imaging was performed using 532 nm pulsed laser source and a wideband polyvinylidene difluoride (PVDF) detector. DOS measurements were conducted using a fiber-optic-based reflectance system. Immunohistochemical (IHC) analysis for CD31 and the hypoxia marker pimonidazole was used for validation. Axitinib treatment resulted in a thirtyfold reduction in the median tumor volume. OA imaging revealed reductions in volumetric vessel fraction and projected vessel area, while DOS showed a transient increase in blood oxygen saturation. IHC confirmed a decrease in microvessel density post-treatment and indicated larger hypoxic areas in treated tumors compared to controls at the experimental endpoint. The newly introduced approach thus facilitates experimental studies aiming at optimization of antiangiogenic treatment regimens and their subsequent combination with other treatment modalities, such as radiation therapy, where effectiveness may strongly depend on the vascular network condition and tumor oxygenation levels.

## Introduction

The ability of malignant tumors to form their own vascular networks is a key factor in their aggressive behavior and the development of distant metastases [1]. Accordingly, the signaling pathways responsible for tumor vascular growth have become critical targets for anticancer therapy. Modern antiangiogenic drugs aim to reduce the supply of oxygen and nutrients to tumors by inhibiting new vessel formation [2]. Their mechanism of action is based on direct prevention of proliferation or migration of endothelial cells in response to angiogenic stimuli or otherwise upon indirect suppression of expression/activity of pro-angiogenic proteins in tumor or stromal cells [3]. Antiangiogenic therapies primarily utilize monoclonal antibodies or small molecule inhibitors targeting tyrosine kinase receptors, including vascular endothelial growth factor (VEGF), fibroblast growth factor (FGF), platelet-derived growth factor (PDGF) receptors, c-Kit, and c-Met. These compounds inhibit receptor activity, disrupting the angiogenic signal transmission that promotes endothelial cell proliferation and migration [4]. Although the use of targeted antiangiogenic drugs has expanded the range of treatable malignant tumors, limitations such as tumor resistance, potential relapses, and severe side effects prevent their wider clinical adoption [5]. Overcoming these limitations involves the development of new drugs and improvement of existing treatment protocols [6]. The impact of antiangiogenic treatments varies depending on dose-time parameters and may result in no detectable changes, normalization of the vascular network (enhancing drug delivery and oxygenation), regression of tumor vessels (reducing nutrient delivery and increasing hypoxia), or toxicity to normal tissues [7]. Therefore, along with the structural state of tumor vessels, the tumor's oxygenation level is considered a key marker of antiangiogenic treatment efficacy [8].

Development of non-invasive methods capable of assessing both structural microvascular changes and variations in tumor oxygenation levels is paramount for preclinical study of promising targeted drug candidates. To assess the vascular alterations, *ex vivo* immunohistochemical analysis using endothelial markers is commonly employed [9]. However, this invasive approach does not permit the monitoring of changes in the neoplasm vessels over time. Several methods exist for intravital, non-destructive examination of the vascular network [10]. Doppler ultrasound can determine the blood flow velocity, but its sensitivity and resolution do not permit the observation of small vessels or those manifesting slow blood flow. The use of angiography with radiolabeled compounds is limited by the method’s low resolution, potential radiation exposure to patients and staff, and nephrotoxicity of the contrast agents. Fluorescence imaging using extrinsically administered contrast agents exhibits a better safety profile, yet it is similarly afflicted with poor spatial resolution in optically diffuse tissues [11]. Another *in vivo* method for assessing the vascular bed of superficial tissues is angiographic optical coherence tomography (OCT), available in either Doppler [12] or speckle correlation [13] modifications.

Optoacoustic (OA) imaging is based on recording ultrasonic pulses generated via light absorption by tissue chromophores. Since living tissues manifest several orders of magnitude weaker scattering of ultrasound waves as compared to light, the OA approach allows for molecular imaging of chromophores with high spatial resolution and probing depth. The strong optical absorption of light by hemoglobin enables numerous applications of OA, particularly in three-dimensional visualization of vascular networks in intact tissues [14]. OA angiography is applicable for studies performed both on laboratory animals and in clinical practice [15].

At present, the invasive polarographic measurement of partial oxygen pressure (pO_2_) remains the most widely used method for determining the oxygen state of tissues. Immunohistochemical studies with endogenous and exogenous markers of hypoxia require *ex vivo* tissue samples and are not suitable for studying biodynamics [16]. Phosphorescent lifetime imaging allows for dynamic studies of oxygen concentration in tissues *in vivo* but requires the introduction of additional dyes - oxygen quenchers [17,18]. Positron emission tomography, using specific radiopharmaceuticals, and BOLD magnetic resonance imaging allow for qualitative assessments of the oxygen state of tissues [19,20] but are not yet widely used due to their high cost, technical complexity, insufficient spatial resolution and the short lifetime of positron-emitting radioactive isotopes.

On the other hand, optical methods have also been used for diagnostics and monitoring of tissue oxygenation levels. Diffuse Optical Spectroscopy (DOS) reconstructs the optical properties of biological tissues – specifically, the absorption and scattering coefficients – detecting light transmission [21]. Spectroscopic measurements then enable the determination of the concentrations of the main tissue chromophores: oxyhemoglobin (HbO_2_), deoxyhemoglobin (HHb), water and lipids. Changes in HbO_2_ and HHb concentrations serve as indicators of oxygen delivery to tissues and its consumption. The content of total hemoglobin (tHb) indicates tissue blood supply, while the ratio of different forms of hemoglobin provides insights into the level of blood oxygen saturation (StO_2_), a parameter that reflects the degree of tissue oxygenation. DOS can be used for assessing the functional state of tumors in both experimental studies and medical practice [22].

To this end, OA and DOS have each been used individually in experimental oncology to study the effects of antiangiogenic therapy on the vascular components of tumors. OA revealed a decrease in hemoglobin content in tumor tissue after treatment with bevacizumab [23]. Additionally, a decrease in the tortuosity of vessels, along with stabilization in their number, was demonstrated when using the DC101 drug [24]. Under the action of trebananib, a decrease in vascular density and an increase in HbO_2_ level have been observed [25]. DOS also indicated a decrease in the tHb level as compared to controls when employing bevacizumab as an antiangiogenic drug [26].

All in all, studies aimed at identifying the structural features of neoplasm bloodstream and its oxygenation level are crucial for understanding how antiangiogenic agents influence the vessel morphology and tumor hypoxia. In this study, axitinib - a clinically approved, multitargeted inhibitor of vascular endothelial growth factor receptor tyrosine kinases VEGFR-1, VEGFR-2, VEGFR-3, FGFR, and c-kit [27] - was used for antiangiogenic treatments. To assess axitinib-induced changes in tissue oxygenation, DOS has been utilized in a reflection mode, while OA was used for high-contrast three-dimensional visualization of the microvascular patterns. Both methods offer a similar depth of investigation and are well-suited for studying subcutaneous experimental neoplasms [28]. The results of the *in vivo* studies were verified by immunohistochemical (IHC) analysis of tumor tissue samples for CD31 and an exogenous hypoxia marker. To the best of our knowledge, it is the first time that this highly complementary combination of *in vivo* methods has been used for studying antiangiogenic effects.

## Materials and methods

### Animals and tumor models

All the experiments were conducted on subcutaneously implanted xenograft model based on the Colo320 colon adenocarcinoma cell line (Colo320DM, ATCC No CCL-220). Twelve female Balb/C nu/nu mice, weighing 20-25 g, obtained from the Pushchino Laboratory Animal Nursery (Russia), were used for the study. The mice were housed under SPF conditions in plastic cages equipped with lids with an air filter, within a ventilated Noroit A-Box 80 cabinet (Noroit, France). A suspension of tumor cells in serum-free RPMI-1640 medium (Gibco, USA) at a concentration of 2 × 10^7^ cells/ml was mixed with Matrigel (Corning, USA) in a 2:1 ratio and injected subcutaneously into the thigh area at a volume of 0.2 ml per mouse.

All animal experiments were conducted in accordance with the European and Russian national guidelines for animal studies and were approved by the Ethical Committee of the N.N. Blokhin National Medical Research Center of Oncology (Protocol No. 04P, dated 29.07.2021).

### OA imaging system and data processing

For visualization, a dedicated OA angiography system [29] was used, based on a diode-pumped laser ONDA532 (wavelength 532 nm, pulse duration 2 ns, maximum pulse energy 0.5 mJ, pulse repetition frequency up to 100 kHz, Bright Solutions, Italy). The 532 nm wavelength is isosbestic for oxy- and deoxyhemoglobin, which allows for correctly assessing changes in total hemoglobin level during tumor monitoring, regardless of blood oxygen saturation. Laser probing of the biological tissue region was performed through a multimode optical fiber FG550LEC (Thorlabs, USA), fixed in the central aperture of the ultrasound detector with a diameter of 1 mm. For signal detection, a broadband (1-30 MHz at -20 dB level) "fisheye" detector based on PVDF (BARI-NN Ltd.) was used with the following parameters: focal distance F = 15 mm, aperture A = 27 mm, angular coverage α = 128°, piezofilm thickness 25 μm. The detector was equipped with a built-in preamplifier featuring a uniform reception band from 1 to 100 MHz and a gain factor of K = 30.

The electrical signals were digitized by a 16-bit analog-to-digital converter CSE25216 (GaGE, USA) with a sampling rate of 200 MHz. The ultrasound detector was placed on a scanning module consisting of two linear stages V-408.132020 (PI micos, Germany). Imaging was performed through an immersion chamber filled with distilled water, with the object imaged through the open top of the chamber. During imaging, the mouse was fixed on a custom platform manufactured by 3D printing using a Sonic Mighty 4K printer (Phrozen, China). The platform featured an opening with a diameter of ≈12 mm for the tumor nodule, which ensured its reliable fixation, especially at late stages of growth.

For laser pulse delivery, a multimode fiber with a core diameter of 500 μm was used. The diameter of the laser beam on the surface of biological tissues was approximately 5.5 mm. The average pulse energy was about 300 μJ. Based on these parameters, the fluence on the tissue surface was estimated to be 1.2 mJ/cm^2^. This value is significantly below the laser safety limit (20 mJ/cm^2^) for the 532 nm wavelength established by ANSI standards.

It has approximately taken 5 min to acquire one 3D image spanning a field-of-view of 10 × 10 × 2 mm. The scanning step was 20 µm.

Reconstruction of the OA data was performed in several stages. First, bandpass filtering in the range of 7-100 MHz was applied to each A-scan [30] to suppress low-frequency noise and enhance the contrast of small vessels, which improved subsequent vascular filtering and binarization. Then, a Fourier reconstruction algorithm was applied to each B-scan (XZ cross-sections) [[31], [32]]. Since the B-scan acquisition rate was at least 3 Hz, the influence of animal motion during a single scan was negligible and did not affect the reconstruction. Next, a motion artifact correction algorithm was applied to the reconstructed B-scans (fast scanning axis): autocorrelation was computed between adjacent B-scans, allowing their depth alignment to compensate for vertical shifts caused by motion. After that, reconstruction was performed in the orthogonal planes (YZ), and the Hilbert transform was applied to the obtained A-scans to extract the positive signal envelopes. During reconstruction, the average effective speed of sound along the path from the sources (vessels in biological tissues) to the detector was used, varying approximately in the range from 1500 to 1510 m/s. For each dataset, the speed of sound was individually adjusted based on the criterion of maximizing the reconstructed vessels sharpness.

The reconstructed OA volumes were further processed for vessel enhancement and quantification in Avizo Software 2022.2 (Thermo Fisher Scientific, USA). The resulting 3D angiographic datasets were batch-processed by a custom workflow in Avizo Software 2022.2 [33] to estimate the volumetric vessel fraction for every angiographic dataset. A volume of interest (VOI) measuring 4 × 4 × 2 mm was selected within the OA dataset, corresponding to the accessible imaging depth and minimum tumor size, and visually verified in three orthogonal projections to ensure that it remained within tumor boundaries and excluded surrounding normal tissue. Preprocessing involved three-dimensional contrast-limited adaptive histogram equalization (3D-CLAHE) with a contrast limit of 2 a.u., followed by application of a Hessian-based vesselness filter optimized for vessel diameters ranging from 40 to 140 µm, using a scale step of 20 µm. Vessel binarization was performed manually using a thresholding approach, with visual control across all images in the series to separate vascular structures from background noise. The threshold was defined using the Avizo auto-thresholding function [33] based on a single highly vascularized control tumor image and then applied to all images in the series. The resulting binary datasets were subsequently subjected to segmentation and skeletonization.

Two-dimensional maximum intensity projections (MIPs) were batch-processed using a custom workflow based on the Particle Analysis toolbox in ImageJ (NIH, USA) to estimate the average projected vessel area. For all images, identical brightness threshold values were applied, selected to highlight the entire tumor-associated vasculature while minimizing background noise.

### Diffuse optical spectroscopy

For the DOS experiments, fiber-optic-based setup [34] in reflectance mode a broadband LED (MCWHF2, Thorlabs Inc., USA) and a spectrometer (QE65000, Ocean Optics Inc., USA) were used as the light source and detector, respectively. The setup includes a probe with four fibers, whose output ends are arranged on a line at equal distances of 1.75 mm from each other; two source-detector distances of 1.75 and 3.5 mm were used in this study. A fiber-optic switch, F-104-03 (Piezosystem Jena, Jena, Germany), is utilized to switch the illumination channels. The absorption coefficient (µ_a_), the HbO_2_, HHb, and tHb concentrations, and StO_2_ were reconstructed from the spectra collected at various source-detector separations across the wavelength range of 520-590 nm [35]. The reconstruction algorithm is based on an artificial neural network. The network was trained using modeled spectroscopic data with various values of oxygen saturation, hemoglobin fraction, and scattering amplitude and power coefficients. A diffusion approximation of the radiative transfer equation in a homogeneous medium was used to create model spectra. With a source-detector separation of 3.5 mm, the maximum probing depth within the specified wavelength range is approximately 2 mm [36]. During DOS investigations, the contact surface of the optical probe was positioned on the tumor surface. For each tumor, 3-6 spectral measurements at different probe positions were performed and averaged.

### Treatment and measurement protocol

**Fig. 1:**
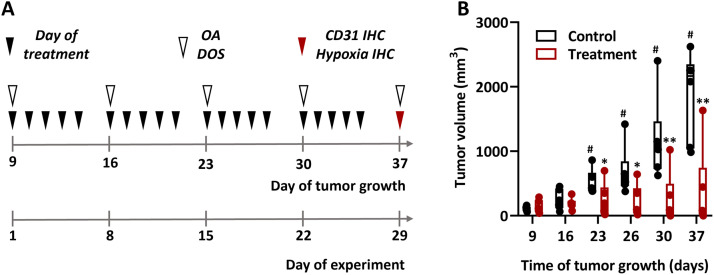
Axitinib inhibits Colo320 growth. (**a**) The experimental timeline: time points of axitinib administration are indicated by black triangles, time points of OA and DOS investigation - by white triangles, time point of morphological study – by red triangle. (**b**) Dynamics of the Colo320 tumor volumes during treatment with axitinib. Individual values, 25-75 percentiles, medians, minimum and maximum of the data set (n = 6). *, p < 0.05; **, p < 0.01 for treated versus control group (Wilcoxon test). ^#^, p < 0.05 for current values versus initial level (Mann-Whitney test).

The experiment timeline is depicted in Fig. 1a. Axitinib (Pfizer Inc., USA) was first dissolved in DMSO, homogenized, and then diluted in saline. The drug was administered *per os* (n = 6) at a dose of 50 mg/kg daily, five days per week for four weeks starting from the 9^th^ day of tumor growth. As a control (n = 6), animals received saline *per os*. OA and DOS studies were conducted on the first day of treatment, prior to its commencement, and every 7 days thereafter. During the OA and DOS experiments, animals were anesthetized using a Zoomed Minor Vet anesthesia-respiratory machine (Zoomed, Russia) with 1.5% isoflurane (Laboratorios Karizoo, Spain) in 100% O_2_ at a gas flow rate of 0.1 l/min. The content of isoflurane and oxygen was chosen based on the study [37], which did not show a significant effect on the level of blood oxygen saturation in normal and tumor tissue of mice. Tumor dimensions were measured every 4-7 days along two perpendicular directions using a caliper, and their volumes were calculated using the formula: *V* = *a* × *b*^2^/2, where *V* is the volume, *a* is the length, and *b* is the width of the tumor node [38].

### Immunohistochemical analysis for CD31

For verification of the *in vivo* OA results, immunohistochemical analysis with antibodies to the mouse endothelial cell marker CD31 was performed for each tumor. On the 37^th^ day of tumor growth (the last day of the experiment), tumors were excised, fixed in 10% formalin (Biovitrum, Russia), embedded in paraffin, and then cut into 4-5 μm thick sections. After dewaxing by sequentially passing through a series of xylene and ethanol solutions and retrieving antigens by keeping in a water bath with citrate buffer (pH=6, 20 min at 97 °C), the sections were placed in distilled water and then incubated with a Peroxidase blocking solution (Abcam, UK) for 10 min at room temperature. They were subsequently washed in PBS and incubated with a Protein blocking solution (Abcam, UK) for 15 minutes. The sections were then incubated with antibodies to the mouse endothelial cell marker – CD31 (Abcam, UK) in a 1/50 dilution for 18-20 h at 4 °C, then washed twice in PBS and incubated with Biotinylated Goat Antibody Anti-Polyvalent Mouse and Rabbit Ab (Abcam, UK) for 30 min at room temperature in humid chambers. After washing the samples twice with PBS, a DAB Substrate solution (Abcam, UK) was applied for 10 min; the sections were then washed in PBS and stained with Mayer’s hematoxylin (Sigma, USA). Finally, the samples were washed in running water, dehydrated by sequentially passing through a series of xylene and ethanol solutions, and placed under a coverslip using a Thermo Shandon Mount (Thermo Fisher Scientific, USA). Microvessels stained for CD31+ were counted in 10 microscope fields per tumor at 200 × magnification using a Nikon Eclipse 80i microscope with a Ds-Fi1 camera (Nikon, Japan), and their numbers were normalized to the area. Areas of necrosis were excluded from the analysis.

### Immunohistochemical analysis for hypoxia

To validate the DOS results, immunohistochemical analysis with the exogenous hypoxia marker pimonidazole (Hypoxyprobe, Inc., USA) was conducted. On the 37^th^ day of tumor growth (last day of the experiment) the animals were intraperitoneally injected with pimonidazole at a dose of 60 mg/kg in a volume of 100 μl of saline, 45 min before euthanasia. *In vivo*, this compound accumulates in tissues with a pO_2_ of less than 10 mm Hg. The tumors were then frozen in a cryotomy medium (O.C.T. compound, Sakura Finetek, Inc., USA), and 2-3 cryosections, each 7 μm thick, were prepared from each tumor. Sections were stained using fluorescein isothiocyanate (FITC)-conjugated anti-pimonidazole monoclonal antibodies. A total of 150 μl of the antibodies, diluted 1:100 in PBS with 1% BSA, were applied to the sections and incubated for 45 min at 37 °C. FITC fluorescence zones across the entire tumor section were identified using a Carl Zeiss LSM 710 laser scanning microscope (LSM) (Carl Zeiss GmbH, Jena, Germany) at 10 × magnification. FITC fluorescence was induced at 488 nm, and detection was carried out across the range of 500-735 nm. The relative hypoxic fraction (RHF) was calculated in Matlab as the percentage of pimonidazole-positive areas relative to the total sample area [16]. The threshold for FITC fluorescence intensity was set based on the signal from tumor sections stained without pre-administered pimonidazole. Areas of necrosis were excluded from the analysis [39].

### Statistical analysis

Measurement data obtained for tumor volume are presented as the box-whisker plots including individual values with 25-75 percentiles, medians, minimum and maximum of the data set. Volumetric vessel fraction and projected vessel area, as well as for CD31+ microvessels, are presented as individual values with means (M) and standard deviations (SD). Data on the relative hypoxic fraction are plotted as individual values with means and standard errors of the mean (SEM). Levels of tHb, HHb, HbO_2_, and StO_2_ are presented as normalized to the baseline individual values with means and SEM. For statistical analysis, GraphPad Prism 8 software was employed. The normality of data distribution was studied by Shapiro-Wilk test. For tumor volume a Wilcoxon test was performed to assess the significance of differences between the current and initial values within the groups and Mann-Whitney test was used to compare untreated and treated groups. For all other parameters a paired t-test was used to calculate the significance of differences between the current and initial values and an unpaired t-test - to compare untreated and treated groups. A statistically significant value was set at p < 0.05. For the comparison of StO_2_ values obtained by DOS and volumetric vessel fraction values obtained by OA with RHF and CD31+ microvessel numbers obtained by IHC, the Pearson correlation coefficient was calculated for the entire tumor cohort as well as separately for each experimental group.

## Results

### Axitinib-induced tumor growth inhibition

Treatment with axitinib effectively inhibits the growth of Colo320 tumors (Fig. 1b). During monitoring period, the median volume of untreated tumors increased approximately 20-fold with p = 0.03.

Conversely, treatment with axitinib significantly inhibited tumor growth; by the 37^th^ day, the tumor median volume in the treated group was 30 times lower than that in the control group (p < 0.01). Notably, one tumor in the axitinib-treated group was not palpable by the end of the experiment. Statistically significant differences in tumor volumes between treated and untreated groups (p = 0.04) emerged starting from the 23^rd^ day of tumor growth (15^th^ day of the experiment).

### Axitinib-induced reduction of tumor vascularity

**Fig. 2:**
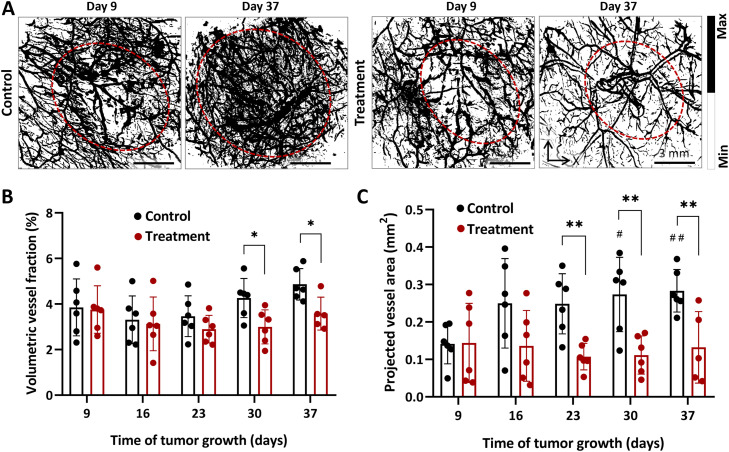
Axitinib-induced reduction of tumor vascularity. (**a**) Examples of OA images of Colo320 vasculature before and after treatment with axitinib. Bar is 3 mm. Dashed lines contour the tumor zones. (**b**) Volumetric vessel fraction of the Colo320 tumors during treatment with axitinib. (**c**) The corresponding projected vessel area. Individual values and M ± SD (n = 6). *, p < 0.05; **, p < 0.01 for treated versus control group (unpaired t-test). ^#^, p < 0.05; ^# #^, p < 0.01 for current values versus initial level (paired t-test).

Treatment with axitinib inhibits the development and alters the structure of vessels in Colo320 tumors. Fig. 2a presents the OA images illustrating the vascular differences between untreated and axitinib-treated tumors at different time points. Images of control tumors depict an increase in vascularity corresponding to the Colo320 cell proliferation. In contrast, images of axitinib-treated tumors show no such increase in vascularization, highlighting the axitinib impact on tumor blood vessel development.

Throughout the monitoring period, from the 9^th^ to the 37^th^ day of the Colo320 tumor growth, the fraction of visualized vessels increased from 3.85 ± 1.25% to 4.87 ± 0.69% (Fig. 2b). The changes were not statistically significant. During this time, projected vessel area (Fig. 2c) increased approximately twofold (p < 0.01).

Treatment with axitinib inhibited vascular growth; the volumetric vessel fraction values remained largely unchanged throughout the experiment and showed statistically significant differences from the control group starting from the 30^th^ day of tumor growth (22^nd^ day from the start of drug administration), with a p-value of 0.02. Furthermore, compared to control tumors, axitinib treatment led to alteration in the vessel structure.

Differences in the averaged projected vessel area values between the groups emerged on the 23^rd^ day and remained until the end of the experiment (Fig. 2c). Post-treatment, a nearly twofold reduction in the averaged projected vessel area values was observed: on the 23^rd^ day, the values were 0.25 ± 0.08 mm^2^ for untreated and 0.11 ± 0.04 mm^2^ for treated tumors (p < 0.01).

### Axitinib transiently enhances tumor oxygen saturation

**Fig. 3:**
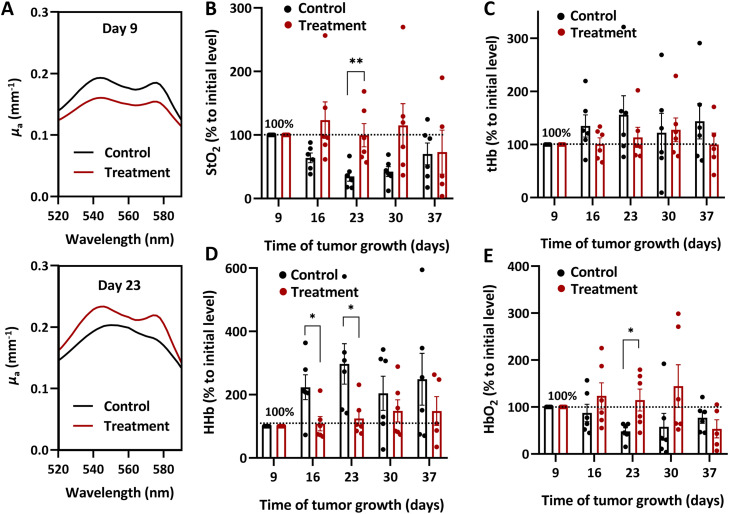
**(a)** Typical spectra of optical absorption coefficient reconstructed with DOS for untreated and axitinib-treated tumors at 9^th^ and 23^rd^ days of tumor growth. Evolution of the normalized StO_2_, tHb, HHb, and HbO_2_ levels is shown in (**b**), (**c**), (**d**), and (**e**), respectively. Individual values and M ± SEM (n = 6). *, p < 0.05; **, p < 0.01 for treated versus control group (unpaired t-test).

Treatment with axitinib transiently increases blood oxygen saturation in Colo320 tumors. Fig. 3a displays absorption coefficient spectra of treated and untreated tumors, obtained using DOS on the 9^th^ and 23^rd^ days of the tumor growth, corresponding to days 1 and 15 from the start of therapy. Before treatment, the absorption spectra of tumors from both groups were similar, indicating a comparable chromophore content. By the 15^th^ day of the experiment, the spectrum shape in control neoplasms had changed, manifesting an increased contribution from HHb. In contrast, the absorption spectra of axitinib-treated tumors exhibited a predominance of HbO_2_.

The dynamics of the StO_2_ level, calculated from the DOS spectral data, are presented in Fig. 3b. In untreated tumors, StO_2_ gradually decreased during the first 15 days of the experiment and remained at reduced levels until the 29^th^ day of the experiment (37^th^ day of tumor growth). Tumors treated with axitinib demonstrated a transient increase in StO_2_ levels compared to the controls. By the 15^th^ day of treatment (23^rd^ day of tumor growth), differences in StO_2_ levels between the groups became statistically significant (p < 0.01). In the subsequent time points of the experiment, no significant differences in StO_2_ were observed between treated and untreated tumors.

In untreated tumors, the decrease in StO_2_ levels was determined by both an increase in the HHb content and a decrease of HbO_2_. Axitinib induced a significant inhibition of the increase in HHb concentration (Fig. 3d), which became statistically significant by the 16^th^ day of tumor growth compared to the controls (p = 0.03). Significant differences in the concentrations of HbO_2_ between the treated and untreated groups were revealed on day 23 of the tumor growth (day 15 from the beginning of drug administration), as shown in Fig. 3e (p = 0.02). No significant differences in tHb concentration were observed between treated and untreated tumors (Fig. 3c), with axitinib causing only an insignificant decrease in the tHb concentration.

### Axitinib decreases the number of CD31 positive blood vessels

**Fig. 4:**
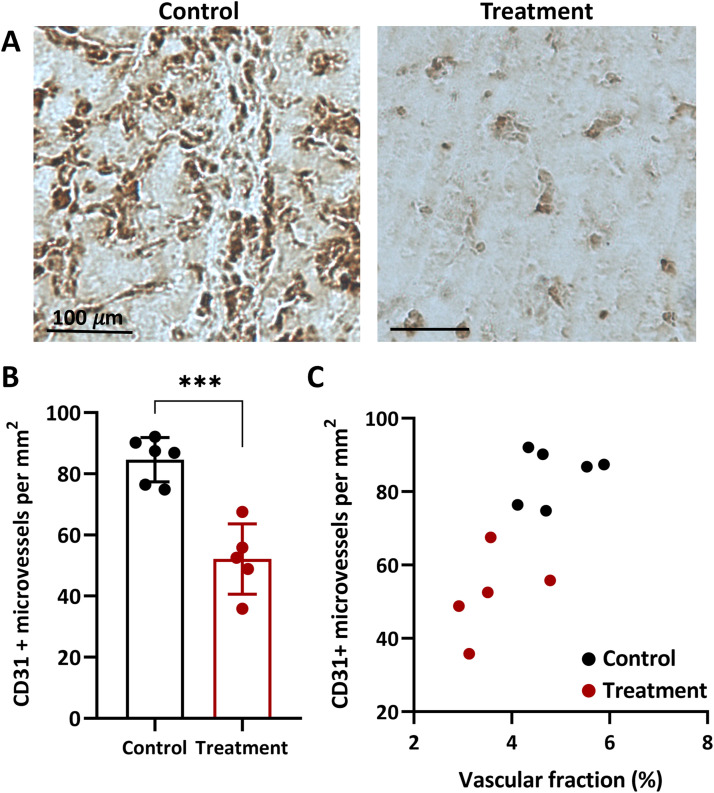
Axitinib-induced decreases of CD31 positive blood vessels. (**a**) Examples of microimages from the tumor sections (scale bar 100 µm). (**b**) The numbers of CD31+ microvessels in the untreated and axitinib-treated Colo320 tumors after immunohistochemical staining for CD31. Individual values and M ± SD. ***, p < 0.001 for treated versus control group (unpaired t-test). (**c**), The values of CD31+ microvessels versus vascular fraction in the treated and untreated tumors.

Treatment with axitinib significantly reduces the number of CD31-positive microvessels in the Colo320 tumors. The microimages of tumor sections after IHC staining and comparison between the number of CD31-positive microvessels in untreated versus treated animals on the 37^th^ day of tumor growth are presented in Fig. 4. IHC analysis showed that in the control group, the number of blood vessels was 84.6 ± 7.2/mm^2^. In the treatment group, axitinib administration resulted in a statistically significant reduction in vessel count to 52.1 ± 11.5/mm² (p < 0.001, Fig. 4a,b).

A comparison of the values of volumetric vessel fraction and the number of CD31-positive vessels reveals a strong positive relationship (Fig. 4c). The data indicate that higher tumor vascularization revealed by OA corresponds to a higher number of vessels as per IHC data. The Pearson correlation coefficient was calculated to be 0.74 (p < 0.01).

### Axitinib increases pimonidazole-positive areas

**Fig. 5:**
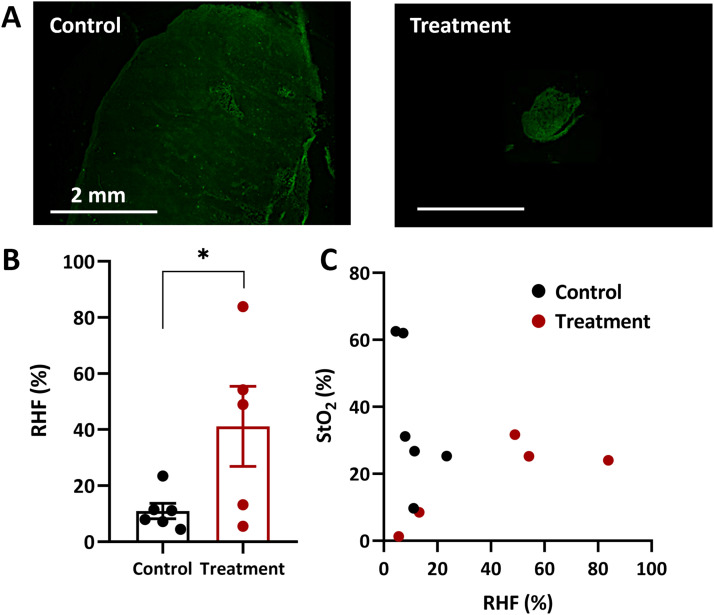
Axitinib-induced increase of pimonidazole-positive areas. (**a**) Examples of LSM images taken from the tumor sections. Bar 2 mm. (**b**) Relative hypoxic fraction (RHF) of the untreated and axitinib-treated Colo320 tumors after immunofluorescent staining for hypoxia with pimonidazole. Individual values and M ± SEM. *, p < 0.05 for treated versus control group (unpaired t-test). (**c**) The RHF values versus StO_2_.

Treatment with axitinib leads to an increase in the hypoxic areas of the Colo320 tumors. Fig. 5 displays LSM images of Colo320 sections after IHC staining for hypoxia in untreated and treated animals on the 37^th^ day of tumor growth. A larger area of brightly colored zones (pimonidazole-positive, hypoxic zones) is characteristic of tumor sections after treatment with axitinib compared to untreated tumors (Fig. 5a). The RHF values of Colo320 after exposure to axitinib were 34.1 ± 9.3%, while in the control group, the RHF values were 10.9 ± 2.7% (Fig. 5b).

A comparison between the StO_2_ values obtained by DOS with the RHF values obtained by IHC indicated a weak correlation (Pearson’s correlation coefficient r = -0.11, p > 0.05). However, a moderate negative correlation was observed for control tumors (r = -0.56, p > 0.05). In contrast, when comparing the results of DOS and IHC for tumors treated with axitinib, a strong positive insignificant correlation (r = 0.81, p > 0.05) was revealed.

## Discussion

Tumor vessels are characterized by series of abnormalities, complicating the delivery of oxygen to the tumor tissue. Additionally, high metabolic activity and rapid proliferation of tumor cells lead to increased oxygen consumption by the neoplasm. Together, these features disrupt the balance between oxygen supply and intake in tumor tissue, leading to the formation of a hypoxic tumor microenvironment [40]. One of the primary molecular mechanisms activated under reduced tissue oxygenation conditions is the stimulation of neovascularization through increased expression of VEGF [41]. As a result, hypoxia stimulates angiogenesis, which in turn promotes further tumor growth and formation of new vessels. A question remains whether such relationships between vascularity and oxygenation persist in the context of treatment with drugs that affect the vasculature of the neoplasm. This issue can potentially be answered by using a combination of methods that allow us to study the dynamics of vascularization and oxygen state of tumors *in vivo*.

Human colon adenocarcinoma cell line Colo320 expresses VEGFR2, one of the target molecules of axitinib [42]. It is characterized by rapid growth, relatively high vascularity and low oxygenation levels [43]. OA imaging demonstrated that, during growth, the untreated tumors exhibited an increase in the fraction of vessels, a typical characteristic of this tumor model, which was accompanied by an increase in their averaged area (Fig. 2). Additionally, tumor growth was associated with a decrease in blood oxygen saturation (Fig. 3), a common feature of many solid tumors. This reduction in oxygenation resulted from an increased level of deoxyhemoglobin, indicating an enhanced oxygen consumption by the tissues, coupled with a decrease in oxyhemoglobin, reflecting reduced oxygen supply to the tissues [44].

We have further shown that axitinib significantly inhibits the growth of Colo320 tumors (Fig. 1b). This drug acts on murine endothelial cells, allowing it to be used to remodel the vascular network of tumor xenografts [45]. Axitinib dosing (50 mg/kg, p.o., 5 days/week) was selected within the reported preclinical efficacy range for oral VEGFR inhibition [46] and is consistent with metronomic regimens used to maintain sustained antiangiogenic pressure [47]. Importantly, angiostatic therapy with axitinib has been reported to induce a transient increase in tumor oxygenation [48], which is directly relevant to our longitudinal OA/DOS measurements.

OA imaging revealed that the inhibited tumor growth is accompanied by reduction in their vascularization as compared to the controls (Fig. 2), suggesting that axitinib prevents the formation of new tumor vessels. The antiangiogenic effects of axitinib have already been confirmed through various *in vivo* and *ex vivo* studies of tumor models. Using MRI and IHC it has been shown that axitinib reduces the number of vessels, decreases vascular permeability, and tumor cellularity [[49], [50], [51]]. This drug demonstrates activity against a wide range of experimental tumors, including renal cell carcinoma, non-small cell lung cancer, glioblastoma, melanoma, and colorectal cancer [46]. In our study using the Colo320 colon cancer model, *in vivo* scanning OA angiography allowed us to observe, with high resolution, the structural changes and distribution of vessels within the tumor node during axitinib treatment over time, as well as to quantify the structural changes.

It should be noted that the volume of interest was selected within the tumor and corresponded to the accessible imaging depth of the OA system. Because the imaging depth was less than 2 mm, the analyzed VOI covered almost the entire treated tumors but only the superficial part of larger control tumors, which may affect interpretation of longitudinal vascular measurements.

Although our OA system has lower spatial resolution than OCT-based angiography [52] – resolving vessels with diameters ≥ 40 µm [29] versus ≥10 µm for OCT – it offers higher imaging depth (≈ 2 mm for OA vs. ≈ 1 mm for OCT) enabling visualization of the vascular network within the full volume of an experimental tumor without creation of skin window chambers.

Axitinib-induced changes of tumor vascular network were accompanied by changes of tumor oxygenation. Certain antiangiogenic drugs may lead to increased oxygenation of tumors [53], including axitinib [48]. According to the “vascular normalization window” theory [7], when specific dose-time parameters of antiangiogenic effects are applied, tumor vessels begin to resemble the structure of normal tissues, and tissue oxygenation level increases. Results of the present study indicate that treatment with axitinib leads to a transient increase in blood oxygen saturation in Colo320 tumors. This response may be related to both enhanced tissue perfusion and a reduction in oxygen consumption by dying parenchymal cells [54]. Based on the DOS data, it can be concluded that the increase in oxygenation of Colo320 tumors is associated with axitinib impact on both oxygen supply and consumption: there is an improvement in oxygen delivery to the tumor (evidenced by increased HbO_2_ level) and a reduction in oxygen consumption by cells (indicated by decreased HHb content). One may thus assume that axitinib not only improves the transport function of blood vessels, but also worsens the condition of the tumor parenchyma. Similar results have been observed in previous studies [25] that examined the effects of trebananib on ovarian xenografts. Therefore, DOS not only assesses changes in tumor oxygenation under antiangiogenic treatment but also elucidates the mechanisms underlying these changes.

It is important to note, that in the present study, anesthesia was maintained with 1.5% isoflurane delivered in 100% oxygen during sequential OA and DOS measurements [37]. It is well established that hyperoxic breathing can influence tissue oxygenation; however, normal and tumor tissues respond differently to increased FiO_2_. In well-perfused normal tissue, switching from air to 100% O_2_ typically results in a marked increase in hemoglobin oxygen saturation [55], whereas tumors - particularly hypoxic regions - demonstrate a markedly decreased response [56,57]. Consequently, hyperoxia may preferentially elevate vascular oxygen saturation in functional vessels without fully reversing tissue hypoxia in poorly perfused tumor regions. In this context, the use of 100% oxygen is unlikely to eliminate intratumoral hypoxia but may increase StO_2_ values in relatively normoxic regions. This should be considered when interpreting the magnitude of observed differences between groups, as hyperoxia may partially amplify contrasts between better-perfused and more hypoxic tumors.

It is crucial to recognize that spectroscopic OA imaging using several excitation wavelengths can independently assess blood oxygen saturation [57]. Previous works have employed several infrared wavelengths to study normalization of vascular bed under the treatment of experimental tumors with the antiangiogenic drug trebananib [25] and sunitinib [58]. However, OA tomography technique traditionally offers lower spatial resolution and does not enable highly detailed visualization of vascular structures compared to OA microscopy. Our setup uses a laser with a wavelength of 532 nm, at which the absorption coefficients of oxy- and deoxyhemoglobin coincide. To perform blood oxygenation measurements, additional wavelengths must be incorporated into the setup. However, this would substantially increase the system's complexity and proportionally extend the acquisition time with the number of laser wavelengths employed. While single-wavelength configuration of our OA imaging system does not permit saturation measurements, we have adopted a dual-modality approach that integrates structural OA imaging of vessels with blood saturation measurements with DOS. Using similar optical wavelengths and illumination geometries in our DOS setup enabled the acquisition of complementary saturation measurements across a volume comparable to that assessed by OA, which has been validated in previous works [28,43]. It is important to consider that OA and DOS investigate partially overlapping, but not spatially identical tissue volumes. In the context of tumor heterogeneity, such spatial discrepancies may affect the direct comparability of measurements obtained by each technique. To account for tumor heterogeneity and the difference in sampling volumes, we performed DOS measurements at 3-6 random points across the tumor surface. The subsequent averaging of the reconstructed values (StO_2_, tHb, HHb, and HbO_2_ levels) provides a representative bulk measurement of the tumor's physiological state. The vascular structure information provided by OA (volumetric vessel fraction and projected vessel area) also represents data averaged over the investigated tumor volume.

The results of OA imaging have been independently validated using standard *ex vivo* methods, such as IHC for the endothelial marker CD31, and α-smooth muscle actin [24,59]. Additionally, DOS data have been verified with pO_2_ measurements and IHC for pimonidazole and carbonic anhydrase IX [[60], [61], [62]]. In our previous work, the combined use of OA and DOS was proven beneficial for studying the natural growth of experimental tumors, validated by IHC for CD31 and hypoxia [43]. In this study, we used a similar approach to confirm the *in vivo* data on tumor vasculature and oxygenation during treatment with the antiangiogenic drug axitinib. IHC data on CD31 indicated a decrease in the number of microvessels when treated with the drug, supporting well the OA data (Fig. 4). Despite the OA being unable to visualize the smallest vessels (under 40 μm in diameter) and IHC accounting for both small and large endothelium-lined vessels, a strong positive correlation was observed between the methods.

Regarding the comparison of StO_2_ values from DOS with relative hypoxic fraction values from IHC (Fig. 5), different trends were identified for treated versus untreated experimental tumors. A moderate negative correlation was observed in untreated tumors, whereas a strong positive but insignificant correlation was found in axitinib-treated tumors. It is important to note that DOS and IHC for hypoxia measure different characteristics of oxygenation: DOS assesses blood oxygen saturation, while IHC quantifies areas of tumor tissue with an oxygen partial pressure less than 10 mm Hg (hypoxic zones). In our experiments, untreated tumors exhibited a direct relationship where lower blood oxygen saturation corresponded to wider hypoxic areas. A similar negative correlation between these parameters was reported in studies of head and neck cancer models [62]. However, in axitinib-treated tumors, high RHF values coexisted with relatively preserved StO_2_. This apparent discrepancy may reflect vascular rarefaction induced by prolonged antiangiogenic therapy. Sustained vessel growth inhibition reduces microvascular density, which may increase the mean distance between perfused vessels and tumor cells, thereby expanding diffusion-limited regions and increase hypoxia [63,64]. Moreover, antiangiogenic therapy may enhance spatial heterogeneity of perfusion, resulting in coexistence of well-oxygenated vessels and poorly perfused or avascular tumor regions. Such heterogeneity can disrupt the relationship between average StO_2_ and the extent of severe hypoxia detected by IHC. Additionally, it is important to consider that pimonidazole, as a circulating drug, may have altered tumor penetration efficiency due to changes in vascular transport function induced by antiangiogenic treatment. These changes could involve aspects such as perfusion or vessel permeability [51]. Moreover, antiangiogenic drugs may reduce interstitial fluid pressure promoting better penetration of medicines into the tumor [65]. Evidences of improved drug uptake by the tumor exist after exposure to axitinib [48,51], which may arguably justify the higher pimonidazole accumulation in axitinib-treated tumors as compared to control. Validating this assumption requires additional experiments to study the delivery and tumor uptake of pimonidazole after treatment with axitinib.

By analyzing the joint results from OA and DOS, we were able to establish the relationship between structural changes in blood vessels and shifts in oxygenation levels during both natural tumor growth and antiangiogenic treatment. Tumor growth is accompanied by an increase in vascularization, which coincides with a decrease in oxygen saturation. It can be assumed that the formation of defective vessels restricted the sufficient delivery of oxygen despite a significant increase in blood supply to the tumor cells. Similar observations were made in our previous studies involving a tumor model based on the Colo320 cell line [43]. Axitinib inhibits the growth of tumor vessels thus effectively enhancing tumor oxygenation. However, such oxygenation improvement appears to be transient, presumably due to a shift in the balance towards anti-angiogenic versus pro-angiogenic factors under the continuous influence of axitinib. It is important to note that, throughout our study, no significant visible changes in the structure of blood vessels were detected upon increasing axitinib dose and exposure time as compared to the baseline. Therefore, by employing a combination of methods that enable the *in vivo* study of tumor vascularization dynamics and oxygen state, it is possible to identify the dose-time interval during which vascular growth is inhibited and oxygenation remains elevated. It suggests that antiangiogenic therapy can effectively be combined with other treatments, particularly radiation therapy, because such enhancement in oxygenation increases the sensitivity of malignant neoplasms to ionizing radiation. The implementation of the “oxygen effect” [66] makes this approach the optimal option for antiangiogenic effects when combined with radiation methods of antitumor therapy [7].

## Conclusion

The combination of optoacoustic imaging and diffuse optical spectroscopy has been demonstrated to effectively monitor vessel structure and changes in blood oxygenation in experimental tumors during growth and antiangiogenic treatment. Under the influence of the vascular endothelial growth factor receptor tyrosine kinase inhibitor axitinib, a decrease in tumor vascularization was observed, along with a temporary increase in tumor oxygenation. *Ex vivo* immunohistochemical analysis for CD31 microvessels showed a high correlation with *in vivo* OA results. Additionally, *ex vivo* IHC for hypoxia corresponded well with *in vivo* DOS results in untreated tumors. However, in axitinib-treated tumors, high pimonidazole accumulation was observed concurrently with high blood oxygen saturation. The proposed combination of *in vivo* methods could be useful for assessing tumor vascular responses to various doses and regimens of antiangiogenic treatment, facilitating their combination with other treatment modalities, particularly radiation therapy.

## Data availability

The data is available from the corresponding author upon reasonable request.

## Funding

Experiments were supported by RSF project 25-15-00238. The development of OA and DOS systems was supported in the framework of the Governmental Project of the Institute of Applied Physics RAS (FFUF-2024-0037). WL acknowledges support from the 10.13039/501100001809National Natural Science Foundation of China (62205089). DR acknowledges support from the 10.13039/501100013362Swiss Cancer Research under grant KFS-5234-02-2021.

## CRediT authorship contribution statement

**Anna Orlova:** Writing – review & editing, Writing – original draft, Visualization, Methodology, Investigation, Funding acquisition, Formal analysis, Data curation, Conceptualization. **Ksenia Akhmedzhanova:** Methodology, Investigation, Data curation. **Anna Glyavina:** Visualization, Investigation, Data curation. **Alexey Kurnikov:** Writing – original draft, Visualization, Methodology, Investigation. **Dmitry Khochenkov:** Writing – review & editing, Writing – original draft, Visualization, Methodology, Investigation, Formal analysis, Conceptualization. **Yulia Khochenkova:** Methodology, Investigation, Formal analysis, Data curation. **Artemii Korobov:** Methodology. **Artur Volovetskiy:** Methodology, Investigation. **Andrey Yudintsev:** Methodology, Investigation. **Anastasiya Nerush:** Formal analysis, Data curation. **Anna Maslennikova:** Writing – review & editing, Writing – original draft, Conceptualization. **Vladimir Vodeneev:** Writing – review & editing. **Ilya Turchin:** Writing – review & editing, Funding acquisition. **Wei Liu:** Writing – review & editing, Funding acquisition. **Daniel Razansky:** Writing – review & editing, Writing – original draft, Supervision, Funding acquisition. **Pavel Subochev:** Writing – review & editing, Writing – original draft, Supervision, Funding acquisition, Conceptualization.

## Declaration of competing interest

The authors declare that they have no known competing financial interests or personal relationships that could have appeared to influence the work reported in this paper.
